# Cell-free circulating tumor DNA analysis for breast cancer and its clinical utilization as a biomarker

**DOI:** 10.18632/oncotarget.20608

**Published:** 2017-09-01

**Authors:** Ru Wang, Xiao Li, Huimin Zhang, Ke Wang, Jianjun He

**Affiliations:** ^1^ Department of Breast Surgery, The First Affiliated Hospital of Xi’an Jiaotong University, Xi’an, P.R. China

**Keywords:** biomarker, liquid biopsy, ctDNA, breast cancer

## Abstract

Circulating tumor DNA (ctDNA) in the blood of cancer patients contains much information on genetic and epigenetic profiles associated with cancer development, progression, and response to therapy. Analysis of ctDNA provides an opportunity for non-invasive sampling of tumor DNA repetitiously and therefore advance precision medicine. Recent development in massively parallel sequencing and digital genomic techniques support the analytical and clinical validity of ctDNA as a promising ‘liquid biopsy’ in human cancer. In this review, we discussed the current status of cell-free ctDNA including ctDNA biology, recently developed techniques for ctDNA detection, breast cancer specific detecting strategies, with a focus on clinical applications of ctDNA-based biomarkers in breast oncology.

## INTRODUCTION

Breast cancer is the most common carcinoma diagnosed among women worldwide, accounting for nearly one in three cancers. It is also the second leading cause of cancer-related deaths in women aged between 35 and 75 years after lung cancer [1, 2]. Due to the improvement in disease management, most breast cancers can be treated by surgery only or surgery and adjuvant therapies including radiation therapy, chemotherapy, endocrine therapy and target therapy. However, as a heterogeneous and dynamic disease, breast cancer exhibits unique acquired somatic mutations and gene expression changes that underpin the two main mortality factors: disease recurrence and drug resistance. Therefore predicting and monitoring response to treatment and disease progression longitudinally is indispensable due to changes in tumor biology and therapy responsiveness over time.

Currently, cancer diagnosis and metastasis monitoring is mainly carried out through tissue biopsy, imaging and/or re-biopsy. Biopsy is a very invasive procedure limited only to certain locations and not always feasible in clinical practice. And imaging cannot give enough information on tumor character to direct further treatment. In order to improve disease monitoring over time and to avoid painful procedure such as tissue biopsy, liquid biopsy may represent a new precious tool [3]. Blood-based circulating biomarkers, including circulating tumor cells (CTCs), cell-free nucleic acids and exosomes, have been studied as ‘liquid biopsies’, that is, surrogate or complementary biomarkers to overcome the drawbacks of invasive tissue biopsies [4]. Increasing number of studies have demonstrated the prognostic value of CTCs in metastatic breast cancer, and there is a burgeoning interest in circulating cell-free DNA (cfDNA), which has a more extensive application in breast cancer management and is more promising in early stage breast cancer due to the development of genetic analysis technologies.

In 1948, Mandel and Métais first identified circulating cell-free DNA as “naked” DNA fragments that is free-floating in the blood or other bodily fluids, and derived from both normal and diseased cells [5]. This attracted little attention in the scientific community due to the low level of cfDNA exits in blood, as well as the difficulty to detect at that time. Interest in the clinical application of cfDNA for medical purposes returned several decades later when scientists first demonstrated that a small percentage of cfDNA originating from the fetus could also be found in the maternal blood, and after that scientists began to explore uses of cfDNA in maternal-fetal medicine [6]. Subsequent investigations revealed cfDNA to be present in higher concentration among patients with inflammatory conditions such as metastatic cancer, trauma, myocardial infarction, autoimmune diseases and sepsis as compared with healthy individuals [7–9].

In 1977, Leon et al. first showed with radioimmunoassays that, on average, cancer patients had an increased amount of cfDNA as compared to healthy patients without cancer [10]. In individuals with cancer, a considerable proportion of cfDNA is thought to origin from normal cells while a small proportion of fragments is represented in the plasma of tumor cells. The tumor specific genetic alterations detected in the primary tumor may also be found in plasma/serum cfDNA of patients with cancer. The fraction of cell-free DNA that contains these alterations of a given patient is named cell-free circulating tumor DNA (ctDNA). Plasma of cancer patients contains ctDNA that carries information on tumor mutation and tumor burden. It has been demonstrated that ctDNA is significantly associated with tumor size, tumor stage, and lymph node involvement in breast cancer [11, 12], and can serve as a biomarker for cancer, with uses from diagnosis to prognosis to monitoring tumor evolution and response to therapy.

**Figure 1:**
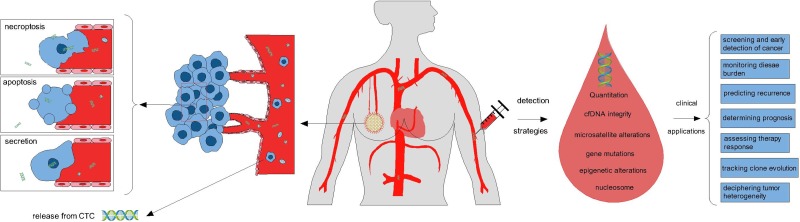
Tumor cells release small fragments of cell-free DNA into circulation by multiple mechanisms, including necroptosis, apoptosis, secretion from tumor cells, and so on Several strategies can be used for the analysis of circulating tumor DNA, such as quantification of cfDNA, detection of cell-free DNA integrity, microsatellite alterations, gene mutations, methylation patterns, and nucleosome. Some potential clinical applications of ctDNA as liquid biopsy to breast cancer management include screening for cancer (potentially as a supplement to mammography), monitoring disease burden, predicting recurrence, determining prognosis, assessing therapy response, tracking clone evolution, and deciphering tumor heterogeneity.

In this manuscript, we reviewed the current status of ctDNA as a ‘liquid biopsy’ in breast cancer, presented a brief introduction of what ctDNA is and current methods for ctDNA detection, and concentrated mainly on the potential application of it in the management of patients with breast cancer (Figure 1).

### Biology of cfDNA

The origin of cfDNA is not fully understood with several possible mechanisms proposed. In patients with tumor, DNA fragments are released by both apoptosis and necrosis in combination with macrophage phagocytosis [13]. It has also been suggested that direct secretion of cfDNA into the plasma is possible [14]. Additional minor source include spontaneous release of newly synthesized nucleic acids, break down of blood cells, break down of pathogens such as bacteria or viruses, and fall off of leukocyte surface DNA [15]. Tumor cells that circulate in the blood, and micrometastatic deposits that are present at distant sites, such as the bone marrow and liver, can also contribute to the release of cfDNA [16]. It has been estimated that for a patient with a tumor that weighs 100 g, which corresponds to 3 × 10^10^ tumor cells, up to 3.3% of tumor DNA may enter the blood every day [17].

Cell-free DNA is a double-stranded nucleic acid with lower molecular weight than genomic DNA that circulates in the bloodstream. It varies from between 70 and 200 base pairs in length up to 21 kb. DNA fragments from necrotic cells have higher molecular weight than fragments from apoptotic cells, a feature which has been exploited for estimation of tumor-derived portion of cfDNA [18]. Nucleic acids are cleared from the blood by the liver and kidney, and they have a variable half-life in the circulation ranging from 15 minutes to several hours [4], making it appealing as a real-time biomarker for assessment of molecular tumor genotype (qualitative) and existing tumor burden (quantitative). Presence of cfDNA is also being assessed in other sources from the body including urine, synovial fluids, saliva and sputum for cancer diagnosis [19].

### Technical approaches for ctDNA detection

The possibility of ctDNA in cancer diagnostics and monitoring comes from the ability to detect the small population of ctDNA from the larger population of normal cfDNA through the identification of tumor-specific (somatic) variations. However, given the fact that ctDNA is diluted by large proportion of wild type cfDNA in the blood and can be contaminated by blood cells easily, techniques for ctDNA analysis are one of the major obstacles in translating ctDNA analysis to clinical practice. A recent development in methodologies allows screening for the presence of ctDNA and brings a new viable tool in early detection and management of breast cancer.

Despite the growing interest in ctDNA analysis in various clinical fields, few studies on sample handling have been reported and no analytical consensus is available. El Messaoudi, S., et al first reviewed articles of cfDNA and focused on standard operating procedure on cfDNA analysis. They examined the preanalytical parameters potentially affecting cfDNA concentration and fragmentation at each pre-analytical step from blood drawing to the storage of cfDNA extracts. Based on the data, they determined the optimal pre-analytical protocols for cfDNA analysis, and ultimately wrote a more detailed guideline of the technical issues of handling and processing cfDNA, including recommendations for translation of cfDNA analysis into routine clinical practice [20].

The common characteristic of cancer is the cancer-specific somatic mutation which represents the main mechanism through which cancer cells overcome physiological cellular signaling pathways (e.g., PI3K/Akt/mTOR, PTEN, TP53) [21–23]. Once cfDNA has been collected, there are two fundamental strategies to analyze: quantification of cfDNA including ctDNA, and analysis of tumor-specific DNA changes including mutations, rearrangements and methylation [24]. For the sake of detecting variant somatic mutations within cfDNA, numerous of methods have been developed. Generally, they can be classified into two approaches: methods based on digital PCR (dPCR) such as BEAMing (beads, emulsion, amplification and magnetics) and droplet digital PCR (ddPCR) and those based on next generation sequencing (NGS) including tagged amplicon deep sequencing (TAm-Seq) [25, 26]. Both approaches have advantages and limitations, and each may find their place in clinical medicine in the future.

### dPCR based methods

The concept of digital PCR was first described in 1992 by Sykes et al. as method assesses individual DNA molecules after serial dilution and/or separation such that the end read-out yields individual reactions with a binary result of either the presence or absence of variant DNA [27]. And until 1999, Bert Vogelstein and Kenneth Kinzler demonstrated dPCR’s ability to detect rare mutations in colon cancer patients [28]. Digital PCR can achieve absolute quantification rather than relative quantification compared to RT-PCR. Thus, point mutations, copy number variations, loss of heterozygosity and aneuploidy can be detected.

BEAMing: is a first-generation dPCR technology combines emulsion PCR with magnetic beads and flow cytometry to identify and quantitate rare genetic molecules found in a larger population of normal or wild type DNA molecules [29].

Droplet Digital PCR (ddPCR): is a variation of emulsion-based dPCR based technology that is sensitive and specific for mutation detection. It removes the need for a reference standard curve as for qPCR. ddPCR is extremely sensitive, able to detect one mutant event in 100,000 wild-type events. However, it is only able to evaluate a limited number of base pair alterations within a single assay. Therefore, ddPCR currently can only be used for hotspot mutation detection, and cannot be applied to mutation discovery [30, 31].

### NGS based methods

NGS, the sequential identification of bases of small fragments of DNA massively and in parallel, has already made enormous contributions to disease research. There is an emerging interest in the field of tumor tissue sequencing for the reason that explicit tumor mutational profile can predict clinical prognosis and direct relevant targeting therapy as tumor evolution. Nevertheless, the traditional NGS approaches is not sensitive enough for detecting ctDNA mutation, where allelic frequencies might easily be less than 1%. As the development of technology, several of the following techniques have been worked out aiming to address this issue and make NGS an option for detection of ctDNA.

TAm-Seq: Tagged Amplicon Deep Sequencing, addresses the issue of sensitivity by adding a targeted amplification step. Forshew et al. first described this method that amplifies and sequences regions from very low allelic frequency ctDNA. They found that this method enriched the sample for the sequence of interest, and therefore increasing the sensitivity as compared to standard NGS [26].

SAFESeqS: is a method that uses single molecule barcoding on one or both strands before PCR amplification, followed by sequencing to reduce sequencing error. In 2011, Kinde et al. first described it. In SAFESeqS, the authors assign a unique identifier—termed “UID”—to each template molecule, which then undergoes amplification, creating a “UID family”. The sample is then sequenced redundantly and data is analyzed to identify true genetic variants [32].

Duplex Sequencing: In this method, Schmitt et al. takes advantage of the double-stranded nature of DNA, independently tagging and sequencing each strand. True mutations should show up in each amplified copy of each strand; single copies of mutations are reflective of errors introduced by PCR or sequencing. This approach theoretically reduces the error rate to 5 × 10^–8^ [33].

Personalized Analysis of Rearranged Ends (PARE): Short tag pairs are applied to ends of template sequences and then mate pairs are analyzed to identify intra- and inter-chromosomal rearrangements. This method can detect genome wide rearrangements and structural alterations in gene regions [34].

In addition to all the methods mentioned above, there are other techniques used for specific detection such as DNA methylation and microsatellite alterations. For instance, modified semi-nested or nested methylation-specific PCR (MSP) for hyper-methylated gene detection reveals high sensitivity and specificity [35–37]. In a word, the rapid development of DNA analysis techniques makes it possible to detect the relatively low concentrations of ctDNA compared to non-tumor cfDNA.

### Detection strategies of breast cancer by ctDNA

Breast cancer represents a heterogeneous collection of diseases with different biological characteristics and clinical outcomes [38]. Genomic alterations play an important role in clone evolution and resistance development during the course of the disease. Since ctDNA is double-stranded nucleic acid that shed from tumor cells into the blood flow, it should contain all the gene information detected in tumor tissue. And as reported, genetic changes such as somatic single nucleotide variants (SNVs), copy number alterations (CNA) and structural variants (SVs) have been detected in ctDNA from breast cancer patients [39–43]. It is these specific mutations help to differentiate ctDNA from normal cfDNA. These somatic mutations are present only in the genomes of cancer cells and are never present in normal cell DNA within the same individual. These features make it possible for elevated specific properties to ctDNA as a biomarker. By taking advantage of features mentioned above, researchers worked out several strategies for breast cancer detection in study filed.

### Quantitation of ctDNA and cfDNA

The initial detection strategy for breast cancer is the quantification of cfDNA to monitor recurrence and metastasis, especially for advanced breast cancer patients. After isolation from the blood, total cfDNA can be measured by fluorescence-based methods utilizing PicoGreen staining or UV spectrometry, or by quantitative real-time PCR with detection by intercalating dyes such as SYBR green or with dual labeled fluorescent/quencher probes [44, 45]. Some quantitative study reported differences in terms of circulating DNA amounts between breast cancer and healthy individuals, more specifically, the plasma DNA concentration in stage II, III and IV of breast cancer were higher when compared with healthy groups [46]. This simple quantitation of the total cell free DNA in blood does not demand high-level technology and can be applied to clinical practice readily. Nevertheless, the diagnostic value of cfDNA quantitation is limited for the significant overlap of cfDNA concentration in normal and cancer patients. Comparing to cfDNA, the level of ctDNA in cancer patients would represent a stable parameter whose fluctuations during the course of the disease may be correlated with clinical outcome. Definitely, it has been demonstrated that high levels of ctDNA correlate with tumor size, lymph node involvement, histopathological grade, and clinical staging [47]. Given the low frequency of ctDNA as compared to cfDNA derived from normal cells, sensitivity is an issue that must be addressed in evaluating ctDNA.

### cfDNA integrity

The ratio of long to short DNA fragments (DNA integrity) is also being studied as a possible biomarker of breast cancer. Taking advantage of the length and ratio of non-coding repetitive DNA sequences such as ALU sequence, we can determine the DNA integrity within cfDNA [48]. The ALU sequences have always been referred to as ‘junk DNA’ in the past; however, scientists gradually realized their importance in various physiological events, such as DNA repair, transcription, epigenetics and transposon-based activity in recent years [49]. These forms of DNA can be detected as cfDNA of different sizes, and different forms such as methylated and unmethylated DNA. Using a PCR assay, studies demonstrates that an ALU DNA integrity assay can be sensitive to detect early stage metastasis to regional tumor-draining lymph nodes, and is associated with response to neoadjuvant chemotherapy in patients with locally confined breast cancer [50, 51]. Studies on these types of cfDNA are still in their initial stage; however, recent studies have shown potential prognostic and diagnostic utility.

### Microsatellite alteration

Microsatellite alterations are another category of genomic changes that we can take advantage of to detect ctDNA in blood, including loss of heterozygosity (LOH) and microsatellite instability (MSI). Tumor-specific LOH analysis of alleles at specific chromosomes of ctDNA can add remarkable diagnostic and prognostic value for early evaluation of primary tumors such as mucosal melanoma, gastrointestinal stromal tumors, hepatocellular cancer, prostate carcinomas, and so on [9, 52–54]. Despite all the encouraging results until now, discrepancies have also been found. These contradictory data from blood samples and tumor tissues attributes mainly to technical problems and the dilution ctDNA in blood by DNA released from normal cells, remind us that the application of this method in clinic still requires further development.

### Breast cancer associated gene mutations

Investigators have previously shown that mutations in proto-oncogenes and tumor suppressor genes found in tumor tissues can be detected in the corresponding blood using the above-mentioned technologies [41]. With the understanding that tumor cells “shed” DNA as ctDNA, and the development of DNA sequencing technologies, numerous of studies have now demonstrated the ability to identify tumor-specific genetic mutations that are patient-specific. Jansen, et al. analyzed DNA from primary tumor and normal tissue and cfDNA from minute amounts of sera by targeted next generation sequencing (NGS) of 45 genes (1,242 exons) in a study. They demonstrates that targeted ion-PGM sequencing of cfDNA is applicable to discover mutations in archived serum samples, and deeper re-sequencing and digital PCR analyses enables more sensitive detection and monitoring of patient-specific mutations in sequential blood specimens [55]. Also, studies have found that good correlation is present between mutations in tumor tissue samples and ctDNA. Higgins et al. used BEAMing to detect PIK3CA mutations in breast cancer patients, and found out the mutations present in approximately 30% of the patients, the results for plasma ctDNA showed 100% concordance to archival tumor tissue when tissue and blood samples were obtained simultaneously [56]. Genes with high mutation frequency in tumor progression such as TP53 and PIK3CA, are paid more attention to because they appear in many tumor subtypes, and therefore, can be detected much easier with relative high sensitivity and specificity. Other clinically relevant mutations which can direct target therapy such as EGFR, KRAS and HER2, also draw much interest in the field of response predictor and resistance monitoring [57].

### Epigenetic alterations

Epigenetic alterations include gene methylation, histone modification, chromatin remodeling and so on [58], they can make a significant impact on tumorigenesis and progression without changing the DNA sequences. Studies examining epigenetic alterations in the plasma of patients with cancer, specifically detection of promoter hypermethylation by methylation-specific PCR, have been performed in various cancer subtypes and hold significant promise as another biomarker of tumor burden and risk assessment [59, 60]. Assays for the detection of promoter hypermethylation may have a higher sensitivity than microsatellite analyses, and have advantages over mutation analyses as well. However, the selection of appropriate tumor-related genes from a long list of candidate genes that are known to be methylated in neoplasia may be the most challenging when applied to clinical practice since there’s no well accepted tumor-specific methylation genes for certain tumor. In breast cancer, Sharma G,et al. analyzed methylation status of a panel of five genes, namely BRCA1, MGMT, GSTP1, Stratifin, and MDR1, and finally found only the methylation status of BRCA1 can be used to monitor response of chemotherapy [61]. Other important methylated genes that have shown prognostic value by ctDNA assays in significant numbers of patients include RAS association domain family 1A (RASSF1A), retinoic acid receptor-b (RARB), and estrogen receptor (ESR1), and all of them are being widely tested in studies around the world [62, 63].

### Nucleosome

A nucleosome is a basic unit of DNA packaging in eukaryotes, consisting of a segment of DNA wound in sequence around eight histone protein cores. Under physiological conditions these complexes are packed in apoptotic particles and engulfed by macrophages [64]. Nucleosome presents in blood flow when there’s too much apoptosis that the excessive nucleosomes cannot be eliminated by macrophages. This can happen both in benign and malignant tumors when tumor cells proliferates fast, or after chemotherapy treatment [65]. Nucleosome can be qualified by enzyme-linked immunosorbent assays (ELISA). As typical cell-death products, the quantification of circulating nucleosomes seems to be valuable for monitoring the response of cytotoxic therapies. Moreover, the outcome of therapy can be predicted by nucleosome levels during the first week of chemotherapy and radiotherapy in patients with lung, pancreatic and colorectal cancer [66–68].

### Clinical application of ctDNA in breast cancer

When focus on breast cancer, the potential applications for clinical practice that stem from ctDNA detection are extensive. This technology has the capability to completely change the paradigm of how clinicians make decisions regarding patient management on adjuvant systemic therapies as well as therapies for metastatic disease. Furthermore, real-time assessment of the molecular profile of a tumor without the need for repetitive biopsies would also help to monitor disease progression and determine rational therapies.

### Diagnosis and screening

Early detection of breast cancer is of leading importance in breast cancer management since early stage patients are regarded as curable and have far good prognosis under today’s treatments. Nowadays, for a definitive diagnosis, mammography, ultrasonography, fine needle aspiration and a tumor biopsy are required. Therefore, a marker that can screen the risk of breast cancer simply would be useful for all people who are at risk of breast cancer.

It has been demonstrated that the median circulating plasma DNA concentration in patients with solid tumors is 17 ng/mL (range: 0.5–1600)—which is 3-fold higher than in healthy volunteers [69]. Therefore, nuclear free DNA in the plasma of cancer patients was introduced as a tool for detection and surveillance of cancer, and many studies have focused on its screening value. To investigate the possibility of using plasma DNA level as the indicator of tumor stage in breast cancer, Agassi R, et al. enrolled 38 patients with breast cancer before surgery, two patients with noncancerous breast lesions, nine patients after surgery, 16 healthy participants, and 29 control women into a study, and measured the cfDNA level by a direct fluorescence assay. They found that pre-surgery patients with cancer had elevated cfDNA levels (1,010 ± 642 ng/mL), which were higher than the other four groups, and the results showed good correlation to stage and enhanced sensitivity to locally advanced disease [70]. cfDNA can be easily detected by fluorescence assay in the blood and the sample preparation are not complicated. However, as we mentioned before, even though there’s difference between cancer patients and healthy people in the mean level of cfDNA, the significant overlap makes it difficult to act as a diagnostic tool. And the low ability to distinguish early breast cancer from healthy patients makes this approach less likely to be clinically useful.

As for ctDNA, the tumor DNA circulating in the blood, is much more specific for oncological application, since the total DNA concentration cannot reflect the cancer type directly. To distinguish ctDNA from noncancerous cfDNA, specific somatic DNA mutations, previously localized in the primary tumor tissue, are identified in the extracted cfDNA, and this relies heavily on the technology development of DNA sequencing. Recent advances in the sensitivity and accuracy of DNA analysis have allowed for genotyping of ctDNA for somatic genomic alterations found in tumors. Breast cancer is considered a kind of disease with high heterogeneity and does not display commonly mutated single loci [71, 72]. In diagnosis setting, patient-specific mutations are not known in advance without sequencing the tumor tissue after biopsy, hence, a considerable sequencing effort of primary tumors is required for the identification of somatic alterations in individual patient to be monitored in plasma. Many genetic alterations are been considered as actionable in breast cancer tumorigenesis and progression, such as mutations of TP53,KRAS, PIK3CA, and promoter methylation of breast cancer-related genes including APC, BRCA1, ER1, GSTP1, HIN1, RARβ, RASSF1 and TWIST [73–75].

In a prospective study, researchers collected 30 primary breast tumors and matched pre- and post-surgery blood samples from early stage breast cancer patients (n=29). Tumors were analyzed by Sanger sequencing for common PIK3CA mutations, and then DNA from these tumors and matched plasma were analyzed for PIK3CA mutations using ddPCR. Results showed that analysis of tumors by ddPCR confirmed all the mutations identified in tumor sequencing and detected five additional mutations. As for plasma samples, Of the 15 PIK3CA mutations detected in tumors by ddPCR, 14 of the corresponding mutations were detected in pre-surgical plasma tumor DNA (ptDNA), and half of the patients had detectable ptDNA after surgery. The study demonstrates accurate mutation detection in tumor tissues using ddPCR, and that ptDNA can be detected in blood before and after surgery in early stage breast cancer patients [76].

Aberrant promoter methylation of genes is a common molecular event in breast cancer. In order to explore the hypothesis that methylation have potential for breast cancer detection, Mohammad O. Hoque, et al. first determined the frequency of aberrant methylation of four candidate genes (APC, GSTP1, Rassf1A, and RARB2) in breast tissues from West African women with predominantly advanced cancer. They used a high-throughput DNA methylation assay (quantitative methylation-specific polymerase chain reaction) to examine plasma from 93 women with breast cancer and 76 controls for the presence of four methylated genes. The results showed that methylation of at least one gene resulted in a sensitivity of 62% and a specificity of 87% in breast cancer detection. Moreover, the assay successfully detected 33% (eight of 24) early-stage tumor [73]. However, results are not always so inspiring in this field. Susan R. Sturgeon, et al. evaluated whether the degree of methylation would lead to a useful serum-based marker of breast cancer by pyrosequencing promoter DNA in a panel of 12 breast cancer-related genes (APC, BRCA1, CCND2, CDH1, ESR1, GSTP1, HIN1, P16, RARβ, RASSF1, SFRP1 and TWIST). And finally draw a conclusion that the modest differences did not provide sufficient ability to distinguish between cases and controls in a clinical setting [77]. This may due to the low sensitive sequencing technology namely pyrosequencing they used in the study. In addition, identification of additional breast cancer specific methylated genes with higher prevalence in early stage cancers would also improve this approach.

### Monitoring disease burden and determining prognosis

Given the fact that there’s no wildly accepted baseline level of ctDNA for breast cancer diagnosis, changes of ctDNA over time do seem to reflect the burden of the disease, determine the prognosis of cancer patients, and help predict therapy response. The management of metastatic breast cancer is extremely difficult for clinicians since each patient has a different situation, and there’s no standard guideline for reference. Therefore, improved biomarkers for tumor burden monitoring to determine the response to treatment are in urgent need. Traditional tumor biomarkers such as cancer antigen 15-3 (CA 15-3) and circulating tumor cells have been widely used in practice. In a recently published proof-of-concept study, ctDNA was shown to be a reliable tool to monitor the tumor burden dynamics of patients with metastatic breast cancer who are undergoing systemic therapy [78]. Dawson, et al. focused on the application value of circulating cell-free DNA carrying tumor-specific alterations and compared it with other circulating biomarkers in breast cancer. They compared the radiographic imaging of tumors with the assay of circulating tumor DNA, CA 15-3, and circulating tumor cells in 30 women with metastatic breast cancer who were receiving systemic therapy. The results showed that ctDNA levels presented a greater dynamic range, and greater correlation with changes in tumor burden, than did CA 15-3 or circulating tumor cells. Among the measures tested, circulating tumor DNA provided the earliest measure of treatment response in 10 of 19 women (53%) [42].

Estrogen receptor α (ESR1) mutations are frequently found in metastatic breast cancer, especially after prior aromatase inhibitor treatment for a period of time. The mutations promote ligand-independent receptor activation and resistance to estrogen-deprivation therapy, but the prevalence of these mutations and their potential impact on clinical outcomes has not been established before. Chandarlapaty, et al. carried out a translational research of BORELO2 trial to determine whether ESR1 mutation is associated with inferior outcomes. By analyzing cfDNA from baseline plasma samples from participants in the BOLERO-2 double-blind phase 3 study, they concluded that of all evaluable patients 28.8% had ESR1 mutations, and the patients with ESR1-mutant ctDNA were poorer prognostic than those without these mutations. The results also demonstrated that the group with D538G mutation derived a similar PFS benefit as wild type from addition of everolimus to exemestane [79]. Consequently, ESR1 mutations are prevalent in ER-positive aromatase inhibitor-treated metastatic breast cancer, ctDNA carrying ESR1 mutation can work as a maker for more aggressive disease biology.

Gene methylation patterns in tumor tissue can be indicative of tumor aggressiveness and likelihood of recurrence, and numerous studies have correlated tissue methylation of individual genes with patient survival [80, 81]. Methylation of the tumor suppressor gene promoter can facilitate tumor progression, for these genes can directly regulate cell growth and metastatic potential. And it can also reflect tumor subtype, which is in turn linked to prognosis. Examples of prognostic methylated genes in serum or plasma include GSTP1 [60], ESR1 [82], RARb2 [83] and so on. RARb2, a retinoic acid receptor, has a complex role in regulating cell proliferation, although it generally plays a role in tumor suppression [84]. In tumors, methylation of RARb2 has been consistently shown to be associated with poor prognosis. For example, Noriko Fujita, et al. developed a one-step methylation specific PCR (OS-MSP) assay to examine the prognostic value of methylated DNA. They take serum samples from 336 primary invasive breast cancer patients and subjected to the OS-MSP assay for the promoter regions of GSTP1, RASSF1A, and RARβ2. Of the 336 stage I/II patients, 33 (10%) were positive for met-DNA in serum and showed a significantly worse overall survival (OS) rate at 100 months (78 vs. 95%; *p* = 0.002) than those with negative findings (*n* = 303) [83]. Hence, detection of target methylated sequences in serum or plasma can be indicative of aggressive phenotype and/or large volume of tumor, both of which correlate with poor prognosis.

### Early prediction of recurrence

As we know, the difficulty in treating late stage breast cancer may be in part because metastatic spread is usually detected only after the deposit has grown large enough to be palpable, cause overt clinical symptoms, or be identified by imaging. That means identification of recurrent disease at the earliest moment will allow for initiation of adjuvant therapies against a smaller tumor burden that has accumulated fewer oncogenic events as soon as possible. From this point of view, earlier detection of recurrence and earlier intervention may bring great benefit for breast cancer patients with high risk. Recent studies have indicated that ctDNA can predict an early recurrence before clinical or radiological recurrence, with sufficient sensitivity and/or specificity. In a retrospective study of 20 patients diagnosed with primary breast cancer, Olsson, et al. identified for the first time that ctDNA monitoring is highly accurate for postsurgical discrimination between patients with (93%) and without (100%) eventual clinically detected recurrence. They also made a conclusion that ctDNA-based detection preceded clinical detection of metastasis in 86% of patients with an average lead time of 11 months (range 0–37 months). By taking advantage of tumor-specific rearrangements in plasma, they established the status of ctDNA as a monitoring tool for early metastasis detection [85]. Garcia-Murillas, et al. conducted similar conclusions by tracking mutations in serial plasma samples for ctDNA. They found that detection of ctDNA in plasma after completion of apparently curative treatment—either at a single postsurgical time point or with serial follow-up plasma samples—predicted metastatic relapse with high accuracy, and mutation tracking in serial samples increased sensitivity for the prediction of relapse, with a median lead time of 7.9 months over clinical relapse [86]. To sum up, continuous monitoring ctDNA with serial follow-up plasma samples after completion of treatment provides much information of genetic events of the subsequent metastatic relapse with high accuracy, making it a monitoring tool for early metastasis detection, therapy modification, and overtreatment avoidance.

### Therapy response assessment

For most metastatic malignancies, a minimum of three cycles of chemotherapy are currently required before treatment response can be assessed based on conventional imaging and biomarkers. This delay exposes many patients to unnecessary toxicity and delays access to other potentially effective therapies [87]. ctDNA can be used to track therapy response including chemotherapy, endocrine therapy, radiotherapy and so on. It is a promising new approach for monitoring response to therapy.

Several studies reported the use of tumor specific mutations to measure ctDNA dynamics at multiple time points during treatment. In a study designed to monitor response to neoadjuvant chemotherapy (NCT) and detect minimal residual disease after surgery, Francesca Riva, et al. used customized droplet digital PCR (ddPCR) assays to track tumor protein p53 (TP53) mutations previously characterized in tumor tissue by massively parallel sequencing. They collected ten milliliters of plasma at 4 time points: before NCT; after 1 cycle; before surgery and after surgery. During treatment, they observed a drop of ctDNA level in all patients but 1, and no patient had detectable ctDNA after surgery. The patient with rising ctDNA level experienced tumor progression during NCT. ctDNA positivity after 1 cycle of NCT was correlated with shorter disease-free (*P* < 0.001) and overall (*P* = 0.006) survival [88]. And in adjuvant settings one could theoretically test each patient post-surgery to determine if there is residual micrometastatic disease in order to make an informed assessment of the need for adjuvant systemic treatment and to prevent the administration of toxic systemic therapies if unnecessary [44]. All the results show that ctNDA has the potential to act as sensitive biomarker to detect minimal residual disease after surgery, and can be an alternative source of genomic information to provide comprehensive data throughout a patient’s clinical course.

Estrogen receptor is the specific target for endocrine therapy, and ESR1 mutations can be selected by prior aromatase inhibitor therapy in advanced breast cancer, influencing the sensitivity of standard endocrine therapies. Are there any relations of ESR1 mutation in ctDNA with therapies including aromatase inhibitor, fulvestrant or PI3K inhibitors? To answer this question, Fribbens, et al. conducted a prospective-retrospective analysis assessing ESR1 mutations in available archived baseline plasma from SoFEA and PALOMA3 trial. ESR1 mutations were found in 25.3% to 39.1% of patients, and patients with ESR1 mutations had improved progression-free survival (PFS) after taking fulvestrant (*n* = 45) compared with exemestane [89]. Takeshita and colleagues investigated the clinical signifcance of sequential measurements of ESR1 mutations in estrogen receptor positive breast cancer patients in a translational research. They found an increase in cfDNA ESR1 mutations from 12 (28.6%) out of 42 MBC patients, and a total of 10 (83.3%) out of 12 MBC patients with increase cfDNA ESR1 mutations showed a poor response to treatment. In survival analysis, increase cfDNA ESR1 mutations may predict a shorter duration of post-endocrine-therapy effectiveness [90]. All these data demonstrate that monitoring of the recurrent ESR1 mutation in ctDNA is a feasible and useful method of providing relevant predictive information either in patient management or in prognosis determination. However, different results are found in a similar study. Researchers assayed hotspot mutations in ESR1 and PIK3CA from ctDNA in clinical trial samples from ER positive metastatic breast cancer patients, and finally made the conclusion that ESR1 mutation allele frequency does not show a consistent pattern of increases during fulvestrant treatment, and progression-free survival is not different in patients with ESR1 mutations compared with wild-type patients [91]. The contradictory results indicate that there are still controversies on the prognostic and predictive value of ESR1 mutation in ctDNA, and additional confirmatory studies with more participates are required in the near future. In spite of this, data from existing research still remind us that ESR1 mutation analysis in plasma after progression after prior AI therapy may help direct choice of further endocrine-based therapy.

Measuring tumor-specific methylation rather than mutation in blood can offer an alternative approach for tracking tumor response to therapy, and similar results can be seen by investigating gene methylation status in circulation. Thomas E. Liggett, et al. explored whether surgical removal of the tumor and subsequent therapy induces changes in plasma DNA methylation, which can also be used to monitor treatment. In this study, samples at three time points were analyzed—before surgery, after surgery and after surgery on tamoxifen therapy, and the methylation of cell-free plasma DNA in 20 breast cancer patients was determined by the previously developed MethDet-56 technique. Researchers have observed differences in methylation of seven promoters (*p* < 0.05) in at least one of the comparisons. Although the number of promoters with changes in methylation varied among the patients, significant changes were observed in all patients [92]. The correlation of methylated circulating tumor DNA (met-ctDNA) with tumor response to neoadjuvant chemotherapy was evaluated in another study by Hiroyo Takahashi and his colleagues. The results showed that in the patients with positive met-ctDNA before neoadjuvant chemotherapy, met-ctDNA significantly decreased after neoadjuvant chemotherapy in those with disease that responded to therapy (*P* = .006), but not in patients whose disease did not respond to therapy. Furthermore, met-ctDNA after NAC was found to be significantly (*P* = .008) correlated to the extent of residual tumor burden [93]. This study again indicates that ctDNA can be used for detection of tumor burden and therapy response, with the hope of identifying patients who need additional treatment earlier and sparing those patients who are cured from unnecessary further treatment.

As described above for determining response of chemotherapy and tamoxifen, ctDNA can also serve to predict radiotherapy response and treatment outcomes. However, few studies to date have explored the application of ctDNA analysis for surveillance after radiation therapy, especially in breast cancer field. In non-small cell lung cancer, Aaron M. Newman, et al. recently provided several examples of using CAPP-Seq to distinguish between normal tissue changes and residual disease treated with fractionated radiation therapy or stereotactic ablative radiotherapy. The results suggest that analysis of ctDNA may have clinical utility by aiding the interpretation of post-radiotherapy imaging studies [94]. Lo et al. showed that for nasopharyngeal carcinoma treated definitively with radiation therapy, plasma EBV DNA levels rose during the first week of treatment (suggesting an increase in cell death) and subsequently declined (suggesting decreasing tumor burden). It is therefore possible that ctDNA changes early during a course of radiotherapy may predict treatment outcome and that early analysis of ctDNA kinetics during treatment could allow clinicians to modify radiotherapy and/or add adjuvant systemic therapy, thus facilitating delivery of truly personalized radiation therapy regimens [95, 96]. The application of ctDNA in tracking breast cancer radiotherapy response is especially promising and should be paid more attention to in further studies.

### Tracking clone evolution and prediction of resistance

A universal challenge in breast cancer treatment is the emergence of resistance to therapy, especially for metastatic breast cancer. Under the pressure of treatment such as chemotherapy, endocrine therapy and targeted therapy, cancer cells can evolve over time, gaining and losing genetic alterations. Repeat biopsies to study clone evolution as a result of therapy are difficult, invasive and may be confounded by intra-tumor heterogeneity. In addition, some metastatic sites are out of reach for biopsy, such as brain metastasis and some visceral metastases deep in the body. On the contrary, the serial analysis of ctDNA can help us track clone evolution and predict the presence of resistance, by simply drawing blood at regular intervals. The results can help direct further clinical decisions in time, and expire patients from ineffective treatment.

Muhammed Murtaza, et al. followed six patients with advanced breast, ovarian and lung cancers for over 1–2 years, and reported sequencing of cancer exomes in serial plasma samples to track genomic evolution in response to therapy. For each case, exome sequencing was performed on 2–5 plasma samples (19 in total) spanning multiple courses of treatment, at selected time points when the allele fraction of tumor mutations in plasma was high, allowing improved sensitivity. Quantification of allele fractions in plasma identified increased representation of mutant alleles in association with emergence of therapy resistance. These results are sufficient to show that exome-wide analysis of ctDNA could complement current invasive biopsy to identify mutations associated with acquired drug resistance in advanced cancers. Serial analysis of cancer genomes in plasma constitutes a new paradigm for the study of clonal evolution in human cancers [97].

### Deciphering tumor heterogeneity

Numerous researches indicate that different tumor cells can show distinct morphological and phenotypic profiles, including cellular morphology, gene expression, metabolism, motility, proliferation, and metastatic potential, namely tumor heterogeneity. This phenomenon can occur within tumors, namely intra-tumor heterogeneity [98]. Furthermore, there is burgeoning evidence to demonstrate that great heterogeneity exists both between primary cancers and metastatic lesions and between metastatic sites within each patient, secondary to evolutionary change. So that primary tumor biopsy or biopsy of one of the metastasis may not completely characterize the genetic profile of metastatic disease [38, 99]. Given that ctDNA is believed to be shed by all tumor sites, it is likely to constitute useful tools to address tumor heterogeneity and therapeutic resistance in both the adjuvant and metastatic settings and better guide therapy.

In a proof of principle study, in order to validate the hypothesis that massively parallel sequencing (MPS) analysis of ctDNA may help define the repertoire of mutations in breast cancer and monitor tumor somatic alterations during the course of targeted therapy, L. De Mattos-Arruda, et al. examined samples in a 66-year-old patient with synchronous estrogen receptor-positive/HER2-negative, mixed invasive ductal–lobular carcinoma with bone and liver metastases at diagnosis by targeted MPS using a platform comprising 300 cancer genes known to harbor actionable mutations. They collected multiple plasma samples during the fourth line of treatment with an AKT inhibitor, and the DNA extracted from archival tumor material and plasma were analyzed. Finally, sixteen somatic non-synonymous mutations were detected in the liver metastasis, of which 9 were also detected in > 5% of the alleles found in the primary tumor sample. Analysis of ctDNA, nevertheless, captured all mutations present in the primary tumor and/or liver metastasis [100]. These findings lend evidence to the idea that ctDNA may constitute an alternative to metastatic lesion sampling for MPS analysis, and may provide a more complete picture of the mutational landscape.

In a similar study, Muhammed Murtaza, et al. presented an extensive comparison of biopsy and plasma samples collected from a patient with metastatic ER-positive and HER2-positive breast cancer receiving two lines of targeted therapy over a 3-year clinical course. They examined archival tumor DNA, synchronous metastasis DNA, and ctDNA collected from plasma over a series of time points of clinical follow-up. The genomic architecture and inferred clonal evolution were characterized by exome and targeted amplicon sequencing. Similar results were collected as the study mentioned above. Their results showed that analysis of ctDNA reflects the clonal hierarchy determined from multiregional tumor sequencing, and provides a dynamic sampling of somatic alterations reflecting the size and activity of distinct tumor subclones and tracks different treatment responses across metastases [101]. This comparison of biopsy and plasma samples in a single patient with metastatic breast cancer shows that ctDNA from plasma identified the dynamic variety of clonal and subclonal tumor heterogeneity, and has important implications to uncover intra- and inter-metastatic heterogeneity and clonal evolution and to establish the use of ctDNA in clinic.

## CONCLUSIONS AND PERSPECTIVES

Numerous genetic and epigenetic alterations are of great importance in carcinogenesis and tumor progression, some of which can also be detected in ctDNA in plasma and serum. The analysis of tumor-specific somatic rearrangements in ctDNA is potentially sensitive and specific to serve as a real-time ‘liquid biopsy’ for the management of patients with breast cancer [4]. The nature of analysis of ctDNA is generally the study of genomics, while ctDNA acts as a new source of DNA sample and provides the possibility of noninvasive assessment of tumor genomes. However, because of the extremely low concentration of ctDNA in blood, it depends largely on the development of new technology with high sensitivity and specificity. In addition, given the fact that to date histological evaluation of tumor tissues obtained from biopsies is the ‘gold standard’ of diagnosis, namely current diagnosis of cancer is basically depend on histology, and up until now the gene alterations for specific cancer diagnosis are not found yet, analysis of ctDNA alone without tissue biopsy is not enough for cancer diagnosis. So that there is still a lot of space for development for the application of ctDNA in diagnosis field in the future. On the other hand, as for cancer screening, prognosis predicting and early detection of recurrence, ctDNA is especially promising since the fluctuation of ctDNA level provides enough information. Furthermore, continuous monitoring of ctDNA can help to track subclone evolution, resistance mutation, and therapy response, which provides abundant information for clinical decisions and studies on mechanisms of tumor progression and resistance developing.

Though studies of ctDNA is promising and draw much attention from around the world, there are, some challenges that need to be overcome for further application. Firstly, there is a great heterogeneity amongst the ctDNA data in different studies using plasma or serum, different technical platforms and patient populations, due to the deficiency of universal standard operating procedure on sample treatment. Pre-analytical parameters potentially affect cfDNA concentration and fragmentation are present at every step from blood draw to the storage of DNA containing sample. Secondly, we described some of the current methods for quantification and detection of a relatively small percentage of mutant or variant molecules within the vast majority of normal or wild-type cfDNA in this review. Though significant progress has been accomplished in the field of ctDNA analysis so far, especially those based on next generation sequencing, continued improvements in technology still needs to be done to guarantee the analytic validity. Moreover, the number of patients included in ctDNA studies is much smaller than the respective number for other biomarker studies such as circulating tumor cell (CTC). For instance, in the ctDNA breast cancer study by Dawson and colleagues [42], only 30 patients were enrolled into the study, whereas the recent CTC study by Rack and colleagues included thousands of patients with breast cancer [102]. Finally, though several studies mentioned above have now partly demonstrated analytical and clinical validity for the application of ctDNA in early and metastatic breast cancer, the studies on clinical utility of monitoring ctDNA is still rare and continues to be controversial. The demonstration of the clinical utility awaits for rigorous prospective clinical trials that designed to direct clinical practice based on the ctDNA results, only through which can we get the information whether patients can truly benefit from ctDNA application.

In conclusion, ctDNA as liquid biopsy has enormous potential, but there are still improvements need to be done before translating into standard clinical tools to assist the management of patients. Ongoing and future studies should pay more attention to the issues mentioned above as we move into the era of liquid biopsies.
